# Self-Limited Focal Epilepsies in Childhood: How Many and How to Treat

**DOI:** 10.3390/pediatric18030074

**Published:** 2026-06-01

**Authors:** Piero Pavone, Francesca Scrofani, Chiara Caruso, Enrico Parano, Agata Polizzi, Raffaele Falsaperla, Antonio Corsello, Giovanni Battista Dell’Isola, Xena Giada Pappalardo

**Affiliations:** 1 Pediatrics, and Psychiatric Department of Child and Experimental Medicine, University of Catania, A.O.U. Policlinico G. Rodolico, 95123 Catania, Italy; ppavone@unict.it (P.P.); agata.polizzi1@unict.it (A.P.); 2 National Council of Research, Institute for Research and Biomedical Innovation (IRIB), Unit of Catania, 95123 Catania, Italy; enrico.parano@cnr.it; 3 Department of Biomedical and Biotechnological Sciences (BIOMETEC), University of Catania, 95123 Catania, Italy; francesca.scrofani@studium.unict.it (F.S.); chiara.caruso@studium.unict.it (C.C.); 4 Department of Medical Science-Pediatrics, University of Ferrara, 44124 Ferrara, Italy; raffaele.falsaperla@unife.it; 5 Department of Clinical-Surgical, Diagnostic and Pediatric Sciences, University of Pavia, 27100 Pavia, Italy; 6 Department of Medicine and Surgery, Saint Camillus International, University of Health Sciences, 00152 Rome, Italy; giovannibattista.dellisola@unicamillus.org

**Keywords:** benign epilepsies, childhood epilepsy, epileptic syndromes, familial infantile seizures, self-limited focal epilepsy

## Abstract

Self-limited focal epilepsies in childhood (SELFEs), formerly referred to as “benign epilepsies in childhood”, constitute a heterogeneous group of epileptic conditions with onset predominantly in the neonatal, infantile, and childhood periods. A defining feature of these syndromes is that seizures arise without underlying structural, metabolic, or other demonstrable cerebral pathology, and the overall clinical trajectory is expected to be favorable, with seizures resolving spontaneously over time. Current nosological frameworks divide SELFEs into two broad categories according to age at onset: (a) neonatal and infantile forms, encompassing self-limited familial and non-familial neonatal, neonatal-infantile, and infantile epilepsies, genetic epilepsy with febrile seizures plus (GEFS+), and myoclonic epilepsy of infancy (MEI); and (b) childhood-onset forms, including self-limited epilepsy with centrotemporal spikes (SeLECTS), self-limited epilepsy with autonomic seizures (SeLEAS), childhood occipital visual epilepsy (COVE), and photosensitive occipital lobe epilepsy (POLE). Despite their historically “benign” label, there is no general agreement to include GEFS + and MEI among the group of SELFEs as both these conditions have been not classified as focal epilepsy in general. Accumulating evidence shows that a subset of affected children subsequently develop additional seizure types, cognitive deterioration, and behavioral or neuropsychiatric difficulties—outcomes that the word “benign” does not adequately communicate. Advances in molecular genetics have identified pathogenic variants affecting ion channels, synaptic transmission, and neuronal excitability, reshaping current understanding of disease mechanisms and phenotypic variability across these syndromes. This review highlights clinically relevant challenges in the diagnosis and management of SELFEs, critically examines emerging genotype–phenotype correlations, and provides evidence-based recommendations for antiseizure medication initiation and withdrawal tailored to individual syndrome characteristics and risk profiles.

## 1. Introduction

The earliest accounts of epilepsies in infants and young children running a benign clinical course are attributed to Fukuyama et al. [1], who provided detailed clinical and electroclinical observations, and to Rett and Teubel [2], who documented neonatal convulsions with a favorable outcome within a single family. Over the subsequent years, in striking contrast to the generally grave prognosis associated with infantile seizure disorders, a growing body of case reports described newborns and infants whose seizures resolved without lasting neurological sequelae [3,4]. Pavone et al. [5] contributed observations on an extended family followed longitudinally over many years, in which four members experienced seizures during the first week of life with no identifiable neurological cause. Treatment with phenobarbital was administered, and after gradual tapering, seizures did not recur beyond four years of age. In a related context, Watanabe et al. [6] described nine infants in whom “benign partial complex epilepsy” was diagnosed through simultaneous EEG-video monitoring. These infants presented with clustered episodes characterized by behavioral arrest, reduced responsiveness, simple automatisms, and mild convulsive activity, with focal paroxysmal discharges on EEG. Both carbamazepine and phenobarbital produced an excellent response, and all children remained seizure-free for over three years. Relatives were noted to have had infantile seizures, yet all achieved normal psychomotor milestones and normal interictal EEGs. This entity was termed “benign partial epilepsy in infancy,” drawing an analogy with the same authors’ previously described “benign partial epilepsy with secondarily generalized seizures” [7].

Renewed clinical interest in childhood seizures with a favorable course emerged in the early 1990s. Vigevano et al. [8] reported a six-month-old infant with benign familial convulsions, and shortly thereafter the same group [9] described five infants aged four to six months whose partial seizures featured head and eye deviation, generalized hypertonus, and bilateral limb jerking with secondary generalization. Interictal EEGs were unremarkable, while ictal recordings showed diffuse discharges originating from the central-occipital region. A positive family history was present, and no further seizures or EEG abnormalities were observed at follow-up. Based on these findings, the authors [9,10] introduced the designation “benign familial infantile seizures” for this clinical entity. Multiple classification systems for neonatal and infantile epilepsies with a favorable course have been advanced over time [11,12]. Building on the preceding International League Against Epilepsy (ILAE) framework, Zuberi et al. [13] stratified neonatal and infantile epilepsy syndromes into two principal groups: (a) self-limited epilepsy syndromes, where spontaneous remission is anticipated alongside normal developmental progression; and (b) epileptic encephalopathies, in which developmental impairment is tied to both the underlying etiology and the ongoing epileptic burden [13]. Syndromes classified within the self-limited group include self-limited (familial) neonatal epilepsy (SeLNE), self-limited (familial) neonatal-infantile epilepsy (SeLFNIE), self-limited (familial) infantile epilepsy (SeLIE), genetic epilepsy with febrile seizures plus (GEFS+), and myoclonic epilepsy of infancy (MEI) [13].

For epilepsy syndromes beginning in childhood, the ILAE classification identifies three major categories; within the self-limited focal epilepsy group, the following syndromes are recognized: self-limited epilepsy with centrotemporal spikes (SeLECTS), self-limited epilepsy with autonomic seizures (SeLEAS), childhood occipital visual epilepsy (COVE), and photosensitive occipital lobe epilepsy (POLE) [11]. This nosological framework was similarly adopted by Manokaran et al. [14] in a recent pediatric-focused review. Menon and Cross [15] enumerate the pediatric self-limited focal epilepsies as including self-limited familial and non-familial neonatal-infantile convulsions, self-limited epilepsy with autonomic seizures, self-limited epilepsy with centrotemporal spikes, childhood occipital visual epilepsy, and photosensitive occipital lobe epilepsy, noting that these conditions share a presumed genetic substrate, age-dependent expression, and typical pharmacoresponsiveness by adolescence. Based on current evidence, the term “self-limited focal epilepsies” (SELFEs) describes a cluster of disorders whose seizures predominate in infancy and childhood, displaying distinctive age-dependent onset, high pharmaco-responsiveness, and a generally favorable natural history with seizure remission over a relatively short interval. A genetic underpinning is presumed, and this group accounts for approximately 25% of all pediatric epilepsies [15,16]. The shift in terminology from “benign” to “self-limited” is justified by the observation that, in 10–20% of cases, the seizures may transition to other epilepsy types, and that cognitive difficulties, neurodevelopmental disturbances, and neuropsychiatric disabilities may emerge over time [17,18]—implications poorly captured by the term “benign” in communication with both families and clinicians. Seizures in childhood serve as clinical indicators of a wide range of disorders, often severe, and carry a substantial psychological burden for affected families. Multiple specialists—neonatologists, pediatricians, neuropediatricians, and neuroradiologists—typically participate in the evaluation of these children. Although the prognosis of childhood seizures, whether favorable or not, is difficult to predict a priori—especially when onset occurs in early infancy—a thorough family and personal history combined with neurological, electrophysiological, with the presence of the generalized discharges and pathogenetic variants including *GRIN2A* mutations, and neuroimaging investigations can provide a reasonable forecast of the clinical course. The objectives of this study were: to individually examine the disorders within the SELFE group; to describe established and emerging genetic variants documented in selected syndromes; and to outline treatment approaches tailored to the differing presentations of each disorder.

The clinical profile of SELFEs (Table 1) has been well characterized and may be summarized under several key features: (a) autosomal dominant transmission, (b) predominant onset in the neonatal, infantile, or childhood period, (c) normal neurological examination, neuroimaging, and laboratory investigations, (d) focal seizures that may secondarily generalize, (e) a positive family history of SELFE or other epilepsy types, (f) prompt response to antiseizure therapy, (g) phenotypic variability across individual syndromes, and (h) a tendency for seizures to remit spontaneously with or without pharmacological intervention [14]. Several epileptic syndromes beginning in childhood and following a favorable trajectory have been identified: SeLFNIE, SeLECTS, SeLEAS, COVE, POLE, GEFS+, and MEI. For GEFS+ and MEI specifically, there is no wide consensus on their inclusion within the SELFE framework, as recent data have documented diverse focal seizure types and neurological complications during these conditions [13]. POLE has been proposed for reclassification within the reflex epilepsy group.

Accordingly, the present PRISMA-guided review aims to map and synthesize the available evidence regarding neonatal-, infantile-, and childhood-onset SELFEs, with particular emphasis on clinical presentation, electroencephalographic characteristics, genetic architecture, natural history, and therapeutic management. Specifically, this review seeks to: (i) summarize the defining clinical and electroclinical features of individual SELFE syndromes; (ii) examine established and emerging genetic variants associated with these disorders and their genotype–phenotype correlations; (iii) discuss current controversies in classification and prognosis, including the debated role of GEFS+ and MEI within the SELFE framework; and (iv) provide an updated overview of treatment approaches and antiseizure medication management tailored to the distinct characteristics of each syndrome.

## 2. Materials and Methods

This review was conducted in accordance with the Preferred Reporting Items for Systematic and Meta-Analyses (PRISMA) guidelines to provide a structured overview of the current clinical, electrophysiological, and genetic evidence regarding self-limited focal epilepsies in childhood (SELFEs). Given the heterogeneity of the included syndromes and study designs, the objective was to map and synthesize the available literature rather than to perform a formal systematic review or meta-analysis. A comprehensive literature search was performed across multiple electronic databases, including MEDLINE, Embase, PubMed, Cochrane Central Register of Controlled Trials, and Scopus, covering the period from January 1996 to January 2025. The search strategy combined controlled vocabulary (e.g., MeSH terms) and free-text keywords related to “self-limited focal epilepsies,” “benign childhood epilepsies,” “SeLECTS,” “SeLEAS,” “COVE,” “POLE,” “GEFS+,” and “MEI.”

**Figure 1:**
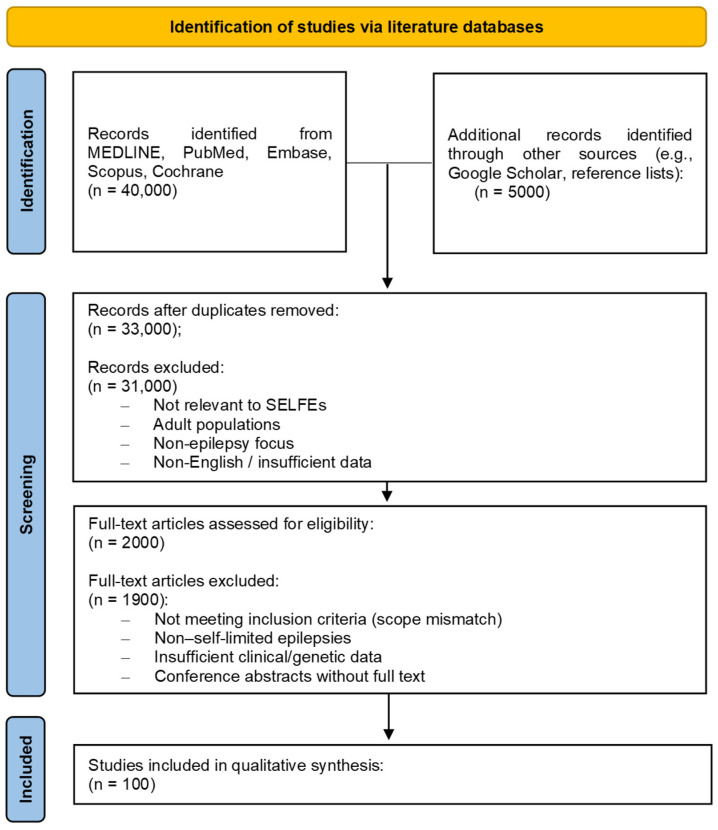
PRISMA 2020 flow diagram illustrating the study selection process for the review.

All retrieved records were exported into a reference management software, and duplicates were removed prior to screening. The study selection process followed a two-step approach: (i) title and abstract screening and (ii) full-text review. Inclusion criteria were defined as studies involving pediatric populations (<18 years) diagnosed with self-limited focal epilepsies or related syndromes, including neonatal, infantile, and childhood-onset forms. Both clinical and genetic studies were considered. Exclusion criteria included non-English articles, studies not focused on SELFEs, adult-only populations, conference abstracts without full text, and articles lacking sufficient clinical or methodological detail. A total of 40,000 records were initially identified across databases and additional sources. After removal of duplicates (*n* = 12,000) and exclusion based on title/abstract screening (*n* = 15,000), around 2000 articles were deemed eligible for full-text assessment. Following detailed evaluation, 100 studies met the predefined inclusion criteria and were included in the final qualitative synthesis, with a particular focus on childhood-onset SELFEs. Data extraction was performed independently by multiple reviewers using a standardized form, capturing study characteristics (author, year, study design), population features, clinical phenotypes, EEG findings, genetic data, and treatment approaches. Discrepancies were resolved through discussion and consensus. Given the nature of the review, no formal risk-of-bias assessment or meta-analysis was conducted; instead, results were synthesized descriptively to map the breadth of available evidence. The study selection process is summarized in a PRISMA flow diagram (Figure 1; Supplementary File).

## 3. Discussion

The clinical characteristics of the SELFEs has been well defined and may be synthetized in several points: (a) an autosomic dominant transmission, (b) prevalent neonatal, infantile and childhood appearance, (c) normal clinical examination, neuroradiologic and laboratory analyses, (d) focal seizures which may evolve to tonic–clonic generalization, (e) positive familial history for SeLFE episodes or other types of epilepsies, (f) rapid response to antiseizure treatment, (g) variability of clinical presentation for each of these disorders, (h) seizures that might tend to disappear with and without the contribution of the antiseizure treatment [14]. Various epileptic syndromes starting in childhood and evolving with a favorable course have been recognized and include SeLFNIE, SeLECTS, SeLEAS, COVE, POLE, GEFS+ and MEI. For the last two, GEFS+ and MEI, there has been no general agreement as to whether they should be inserted into the group of SELFEs, as recent observations have reported new types of focal seizures—neurological complications in the clinical course of the disorders. POLE has been included in the group of the Reflex Epilepsy. 

### 3.1. SeLFNIE

In this condition, the terms “convulsions,” “epilepsies,” and “seizures” have been used interchangeably. The word “convulsions” may be more apt for neonates and young infants, since abnormal movements such as jerking, pedaling, and stepping observed in the first weeks of life are not always considered to be epileptic in nature [15]. Zuberi et al. [13] further subdivided the early-onset SELFE group based on timing of seizure onset, distinguishing self-limited (familial) neonatal, neonatal-infantile, and infantile epilepsy syndromes. Although these subtypes differ in their precise age of onset, they share enough clinical characteristics to be discussed collectively here. In the neonatal subgroup, seizure onset typically occurs between the second and fifth day of life, whereas in the infantile subgroup it falls within the first weeks to months. Episodes are focal, consisting of motor tonic or clonic activity alternating between sides, and are sometimes accompanied by non-motor features such as cyanosis and apnea. Episodes are generally brief (lasting only a few minutes), and tend to occur in clusters, occasionally recurring after a brief apparent remission. Between episodes, affected infants are alert and behaviorally normal. The interictal EEG background is reported as normal, while ictal recordings may reveal focal discharges predominantly arising from temporal or posterior regions. Seizure remission typically occurs between 6 weeks and 24 months of age [13,17]. Family histories in these children often reveal neonatal-infantile SELFEs as well as diverse seizure types among relatives. Phenobarbital is considered the first-line antiseizure medication (ASM) regardless of etiology, with phenytoin or carbamazepine serving as first-line options in confirmed channelopathies [19]. Gene variants associated with this group include *KCNQ2*, *KCNQ3*, *SCN2A*, *SCN8A*, and *PRRT2* [17,20,21].

A SeLFNIE family was the subject of extended clinical observation spanning many years [5]. We recently had the opportunity to re-contact one of the originally affected members, who was a neonate at the time of the initial report and is now 43 years old. He reported being in good health, and neither he nor his two children (aged 8 and 11 years) have experienced any neurological problems to date. *KCNQ2* mutations have been identified in children with a wide range of clinical severity, from otherwise healthy neonates belonging to the self-limited (familial) neonatal epilepsy group (*KCNQ2-SeLNE*) to the severe end of the spectrum represented by neonatal-onset developmental and epileptic encephalopathy (*KCNQ2-NEO-DEE*) [22]. The severe phenotype manifests in the first week of life with frequent focal motor tonic and clonic seizures, interspersed with autonomic signs, and showing poor pharmacological control. EEG typically demonstrates burst-suppression or multifocal epileptiform activity. Pavone et al. [5] described a child who presented on day 3 of life with multifocal clonic seizures in clusters, migrating alternately across both body sides and lasting only briefly. Autonomic features were also present, including cyanosis, ocular deviation, and vomiting. The ictal EEG on day 3 showed a chaotic background, whereas the interictal EEG on day 7 was normal, as was the clinical examination and laboratory workup. Genetic analysis identified a pathogenic variant c.1508C>G in heterozygous state in *KCNQ2*. The infant responded well to phenobarbital, with seizure remission achieved; treatment was discontinued at four months of age. Different epilepsy phenotypes were documented among other family members.

### 3.2. SeLECTS

This syndrome is also referred to as “benign epilepsy with centrotemporal spikes” (BECTs) and benign Rolandic epilepsy (BRE). It occurs in children with otherwise normal cognitive and neurological development and is widely regarded as the most prevalent epileptic syndrome of childhood, with an estimated incidence of 10–20 cases per 100,000 children under 15 years of age [23,24]. The seizure focus characteristically lies within the Rolandic region, which spans the frontal (motor) and parietal (sensory) cortices, producing facial, oral, and pharyngeal motor and sensory manifestations in affected children. Seizure onset spans ages 3 to 13 years, with a peak at 9–10 years [24]. Clinically, episodes are brief (typically 2–3 min), infrequent, and focal, presenting as hemifacial motor activity often accompanied by somatosensory symptoms. Facial twitching and stiffness, along with numbness or tingling affecting one side of the face, throat, tongue, lips, gums, inner cheek, and teeth, are frequently reported, giving rise to drooling, gurgling, and hypersalivation. Focal seizures can spread to bilateral tonic–clonic convulsions. Symptoms predominantly emerge shortly after sleep onset or in the peri-waking period. Children with onset before the age of 5 years appear more likely to experience a longer duration before seizure and EEG resolution. The EEG background is normal, with typically unilateral high-amplitude centrotemporal sharp waves occurring in repetitive bursts, sometimes followed by a slow component; these discharges are enhanced during drowsiness and sleep. In children experiencing only isolated nocturnal seizures, antiseizure therapy is generally not considered necessary [25]. When treatment is warranted, seizures typically respond well [26], and a single bedtime dose of levetiracetam has been demonstrated to be effective in a randomized clinical trial by Fan et al. [27]. Neuropsychiatric comorbidities have been documented in SeLECTS: Ross et al. [28] identified an elevated risk of neuropsychiatric symptoms in affected children over the course of the disease. Academic underachievement and behavioral problems [29], as well as dyslexia and dyscalculia [30], have also been reported. Lacey et al. [31] found a higher prevalence of neurodevelopmental comorbidities and school difficulties in Welsh children with SeLECTS compared to the general pediatric population, attributing this to disruption of functional brain networks mediated by centrotemporal spiking activity [31]. Persistent increased functional connectivity has been observed during sleep, with further elevation during and around spike events [32]. Atypical presentations of SeLECTS—including status epilepticus, electrical status epilepticus during slow-wave sleep, and Landau–Kleffner syndrome—have been described [33]. Certain features, such as comparable age at onset, an overall favorable prognosis, and occasional co-occurrence in the same child, are shared between childhood absence epilepsy and SeLECTS [34]. Gene variants documented in SeLECTS include those in *KCNQ2* and *KCNQ3* [35], *ELP4* [36], and *GRIN2A* [37].

### 3.3. SeLEAS

This disorder was first delineated as a distinct syndrome by Panayiotopoulos in 1989 [38]. Previously designated as Panayiotopoulos syndrome and early-onset benign occipital epilepsy, it is an idiopathic, age- and self-limited condition that ranks as the second most common focal epilepsy of childhood. The disorder predominantly affects children between 3 and 6 years of age, with a broader range of 1 to 14 years [38,39]. A history of febrile seizures is present in 5–7% of cases. Its clinical signature consists of three cardinal ictal features: nocturnal onset, tonic ocular deviation, and emesis [38], reflecting the prominence of focal autonomic manifestations. Seizures are often preceded by a transient alteration of consciousness and consist of prolonged episodes of nausea and vomiting, pallor, urinary incontinence, hypersalivation, pupillary dilatation, and unresponsive flaccid loss of postural tone (ictal syncope), lasting up to 30 min. Although they can arise at any point during the day, they occur most frequently during the initial phase of sleep [40,41]. Episodes may extend from several minutes to more than two hours, occasionally evolving into autonomic status epilepticus, with spontaneous remission typically occurring within a few years of onset [40,41]. Ictal events can closely mimic non-epileptic conditions such as syncope, migraine, cyclic vomiting syndrome, motion sickness, sleep disorders, gastroenteritis, and acute encephalitis [41]. Caraballo et al. [42] reported on 192 subjects, noting a peak age of onset at 5 years. Autonomic features, including eye deviation, dominated the ictal semiology. In virtually all patients, seizures occurred during sleep, though in approximately one-third they were also observed during wakefulness. The overall prognosis was good: 84 patients (44.2%) had only a single seizure, 79 (41.2%) had 2–3 episodes, and 28 (14.6%) had multiple events. Generalized evolution of seizures was common, and roughly one-third experienced partial status epilepticus. In the series of Durá-Travé et al. [43], which included 37 patients, the mean age at diagnosis was 5.4 years and a female-to-male ratio of 2:1 was observed. Autonomic features or vomiting were reported in 70.1% of patients, and ictal eye and/or head deviation was frequently noted, as was progression to partial or generalized seizures. Seizure recurrence occurred primarily within the first six months after diagnosis, with 82.9% of patients remaining seizure-free beyond two years from onset. Syncope-like epileptic seizures were documented by Koutroumanidis et al. [44] in at least one episode among 17 of 33 children (51.5%).

As reported by Panayiotopoulos et al. [38], the EEG background is normal. Interictal recording typically reveals multifocal high-voltage repetitive spikes and slow waves with a predilection for the occipital regions, though anterior, central, temporal, and midline involvement may also occur. Ictal EEG is generally unilateral and frequently shows posterior onset with rhythmic slow activity intermixed with small spikes [38]. Oguni [45] emphasized that the morphology, distribution, and appearance of interictal EEG foci may vary with the child’s age at the time of recording; additionally, the author proposed that the absence of frontoparietal EEG foci may serve as a specific marker supporting the diagnosis [45]. Psychomotor development is usually preserved, and the long-term outlook for both seizure frequency and evolution is generally favorable. Caraballo et al. [46] described atypical outcomes in two children: a 3-year-old who developed frequent inhibitory seizures causing pseudoataxic gait, behavioral disturbances, and aphasia, and a 9-year-old girl who, after 18 months of seizure freedom, developed mild intellectual disability. Mild deficits in language and executive functioning, as well as poor academic performance, have also been documented in this disorder [47].

To our knowledge, no gene mutations specifically linked to this disorder have been identified. However, familial clustering in siblings and across generations suggests a polygenic basis. There is general consensus that treatment should be guided by seizure frequency and severity, with oxcarbazepine, carbamazepine, levetiracetam, lamotrigine, and sulthiame being the most frequently employed antiseizure agents [15].

### 3.4. COVE

This syndrome was previously designated as idiopathic childhood occipital epilepsy—Gastaut type, and as late-onset (benign) childhood occipital epilepsy. It is distinguished from other forms of benign occipital epilepsy by its distinct clinical presentation. COVE is a relatively uncommon syndrome manifesting with frequent and brief focal sensory episodes—typically lasting from a few seconds to fewer than three minutes—characterized predominantly by visual phenomena occurring in the awake state and often followed by headache with migrainous features [48]. Elementary visual hallucinations, ictal blindness, and post-ictal headache are among the most commonly reported symptoms [49]. Onset can occur between ages 1 and 18 years, with the condition most commonly presenting in late childhood or early adolescence, peaking at 8–9 years. The ictal semiology includes visual hallucinations, transient blindness, orbital pain, and occasionally focal sensory or motor disturbances [50]. Visual phenomena are characteristically described as small, multicolored circular shapes appearing in the peripheral visual field, which progressively expand toward the center and may spread horizontally, sometimes followed by eye deviation. Caraballo et al. [51] reported on 33 patients with COVE and identified a mean age at onset of 8.5 years. Visual manifestations were the predominant ictal feature, with eye deviation being the most frequent symptom; episodes occurred predominantly in the awake state, though less commonly during sleep as well. A subsequent publication [52] provided further differentiation of COVE from migraine and other epilepsy types. Wakamoto et al. [53] reported on 12 affected children (three boys, nine girls) with a median age of onset of 10.3 years. The most frequent ictal manifestations included elementary visual hallucinations (75%), blindness or blurring of vision (50%), and secondary generalized tonic–clonic seizures (58.3%). A frequent association with generalized tonic–clonic seizures and atypical evolution from childhood absence epilepsy was also documented [53]. Interictal EEG showed occipital spike-wave paroxysms responsive to eye closure and opening in all patients; extra-occipital spike-wave activity was present in four patients (33.3%) and generalized spike-wave discharges in two (16.7%). In a retrospective analysis of 129 patients with childhood occipital epilepsy of Gastaut (COE-G), Verrotti et al. [54] reported visual hallucinations as the initial symptom in 62% and the sole manifestation in 38.8% of cases, with an overall favorable prognosis and good response to antiepileptic therapy. A more recent study [55] of 30 patients diagnosed with COVE between 1988 and 2023 found elementary visual hallucinations, such as colored lights, in 93% of cases; orofacial seizures were recorded in 7%, and nocturnal seizures in 37%. EEG abnormalities were primarily occipital and resolved over time in 85% of patients. At final follow-up, 77% had achieved seizure freedom and 47% had discontinued medication [55]. Shu et al. [56] observed a correlation between occipital spike-wave discharges and the state of eye closure, noting that EEG abnormalities were present with eyes closed and suppressed upon eye opening. In their cohort of seven patients, two continued to experience frequent seizures despite treatment, with associated learning difficulties and behavioral disturbances. A distinctive EEG feature in COVE, as described by Rots et al. [57], is fixation-off sensitivity—the elicitation of epileptiform discharges when visual fixation is removed. The long-term prognosis of COVE is generally favorable, with seizure remission and EEG normalization expected by the late teenage years [49], and an excellent outcome reported in approximately 80% of cases [51]. The genetic architecture is considered complex and polygenic.

Fonte et al. [48] recently described a mother-son pair displaying clinical and EEG features of COVE/POLE in whom a large deletion at 5q34 encompassing the entire GABRA1 gene and a portion of GABRG2 was identified. Antiepileptic treatment is indicated for children with frequent ictal events; carbamazepine has demonstrated efficacy in approximately 90% of treated cases [50].

### 3.5. POLE

POLE is an uncommon condition defined by focal visual seizures triggered by photic stimuli. It was previously termed idiopathic photosensitive occipital lobe epilepsy and belongs to the broader category of reflex epilepsies (RE)—a group of epileptic events consistently and reproducibly provoked by specific stimuli such as flickering light or reading [58]. Seizure onset in POLE spans ages 4 to 17 years (mean 11 years, range 1–50 years) and shows a marked predominance in females. Light sources such as television and video games are the principal seizure triggers. Episodes are brief (fewer than 3 min) and variable in frequency, featuring visual sensations including colored spots, formed visual hallucinations, or visual blurring and loss of vision that migrate across the visual field. Symptoms may progress to include autonomic features, impaired consciousness, and evolution to generalized tonic–clonic seizures. Sirin et al. [59] reported on 29 patients with POLE (mean age 20.1 ± 7.6 years) and noted overlapping features with genetic generalized epilepsy in approximately one-third; this overlapping subgroup showed higher rates of febrile seizure history and self-induction behavior. Guerrini et al. [60] studied 10 patients (8 female, 2 male) with idiopathic photosensitive occipital lobe epilepsy, ranging in age from 8 to 30 years (mean 17 years). Seizure onset was reported between ages 5 and 17 years (mean 11 years); stimulus-related episodes began with elementary visual symptoms and progressed to a gradual clustering of headache, epigastric discomfort, and vomiting, with either preserved or mildly impaired responsiveness. EEG features included normal background activity, occipital spikes and waves, and a photoparoxysmal response appearing in the occipital area, in a generalized distribution, or both. Politi-Elishkevich et al. [61] described 16 children and adolescents, including two sets of siblings and seven patients with a positive family history of seizures. All patients had occipital-onset seizures, and 15 showed secondary generalization to tonic–clonic seizures; seizure frequency was relatively low in all but one patient. Two patients subsequently developed myoclonic seizures [61]. Remission is observed in most cases by puberty. Trigger avoidance and use of tinted glasses may be of benefit.

Despite the electroclinical differences among individual syndromes, several unifying themes emerge across the SELFEs spectrum. Most disorders share an age-dependent onset, focal seizure semiology with possible secondary generalization, normal neurological examination and neuroimaging, characteristic but often evolving EEG abnormalities, and an overall tendency toward spontaneous remission during childhood or adolescence. However, increasing evidence demonstrates that seizure remission does not invariably correspond to a completely benign neurodevelopmental outcome. Cognitive, behavioral, language, attentional, and psychiatric comorbidities have been documented in multiple SELFEs, particularly in SeLECTS, SeLEAS, GEFS+, and MEI, suggesting that these disorders may reflect transient dysfunction of developing cortical networks rather than isolated epileptic phenomena alone. This evolving perspective has important clinical implications because treatment decisions should not rely exclusively on seizure frequency but also consider neuropsychological profile, sleep-related EEG abnormalities, school performance, and quality of life.

### 3.6. Genetic Epilepsy with Febrile Seizures Plus (GEFS+)

GEFS+, formerly termed “generalized epilepsy with febrile seizures plus,” is a genetic syndrome with a broad phenotypic spectrum affecting multiple family members and encompassing a variety of presentations ranging from typical to atypical febrile seizures and/or other seizure types, including absences, myoclonic seizures, atonic seizures, myoclonic-astatic epilepsy, and, uncommonly, severe epileptic encephalopathy [62,63]. The qualifier “generalized” was subsequently removed from the syndrome name when it became apparent that focal features were also prevalent among affected family members [62]. GEFS+ was formally incorporated into the ILAE syndrome classification as a recognized entity in 2001 [62]. The original description is attributed to Scheffer and Berkovic [64], who characterized a large family in which related members across four generations exhibited varied seizure phenotypes: some presented with atypical febrile seizures (FS+) with either unusually early onset (median age 1 year) or late persistence beyond 6 years of age and multiple recurrences, while others displayed combined phenotypes such as FS+ with absence, FS+ with myoclonic seizures, FS+ with atonic seizures, and myoclonic-astatic epilepsy [64]. The etiology is complex, reflecting a heterogeneous genetic predisposition that interacts with febrile stimuli, including specific viral infections [65]. Autosomal dominant inheritance with incomplete penetrance is the most observed pattern, although other inheritance modes have been reported [66]. Thomas et al. [67] studied 80 recruited families with the aim of better characterizing both definite and borderline GEFS+ phenotypes, identifying four broad subtypes: classical GEFS+, borderline GEFS+, unclassified epilepsy, and alternative syndromic diagnoses. The borderline GEFS+ group shared many characteristics with classical GEFS+ but included a higher proportion of adults with focal rather than generalized seizures and a twofold excess of migraine [67]. To refine the phenotypic spectrum further, Zhang et al. [68] analyzed 409 affected individuals from 60 families and identified focal seizures without preceding febrile seizures in 16/409 (4%), classic genetic generalized epilepsies in 22/409 (5%), and afebrile generalized tonic–clonic seizures in 9/409 (2%). Pathogenic variants in established GEFS+ genes were identified in approximately one-third of tested families. A detailed clinical and genetic analysis of GEFS+ was conducted by Chen et al. [69] in 392 affected individuals from 133 families. The predominant phenotypes were febrile seizures (FS) (288/392, 73.5%) and FS+ (70/392, 17.9%); other phenotypes included FS/FS+ with generalized seizures (21/392, 5.4%), FS/FS+ with focal seizures (8/392, 2.0%), Dravet syndrome (8/392, 2.0%), afebrile generalized tonic–clonic seizures (17/392, 4.3%), complex phenotypes (5/392, 1.3%), and unclassified seizures (35/392, 8.9%). Regarding the role of fever sensitivity, patients with lower thermal sensitivity were more likely to exhibit focal, myoclonic, and tonic seizures and to have multiple seizure types, whereas those with high thermal sensitivity more often had a single seizure type. Genetic testing of GEFS+ patients most frequently yielded variants in voltage-gated channel genes *SCN1A*, *SCN1B*, *KCNT1*, *CACNA1A*, *CACNAIH*, and *GABA* receptor-related genes *GABRA1*, *GABRB2*, *GABRB3*, and *GABRG2* [69]. Baso et al. [70] proposed predictive criteria for evolution to epilepsy or GEFS+ in children presenting with FS, FS with status epilepticus, or recurrent FS: in a cohort of 231 children, a family history of epilepsy, higher total number of seizure episodes, older age at FS onset, and the occurrence of afebrile seizures were each independently associated with an increased risk of progression. Genetic variants associated with GEFS+ have additionally been reported in *KCNAB3* [71], *GABRG2* (Q390X) [72,73], and *SCN1A* (A2336G, exon 13) [74]. Diagnosis rests on the seizure type and a comprehensive family history. EEG shows a normal background with generalized discharges. Seizures tend to remit by late childhood or puberty. A significantly elevated rate of auditory phoneme discrimination impairment, articulation difficulties, and language disorders has been reported in patients with GEFS+ [75].

### 3.7. MEI

Myoclonic epilepsy of infancy (MEI), originally termed “myoclonic epilepsy of childhood” by Jeavons [76] and subsequently described as “benign myoclonic epilepsy in infants” by Dravet et al. [77,78], is an uncommon idiopathic generalized epilepsy presenting with brief myoclonic seizures in previously healthy and normally developing children, with onset confined to the first three years of life. The term “benign” has been abandoned given the recognition that the course is not invariably uncomplicated; the disorder is now classified within the idiopathic generalized epilepsies with age-related onset [11]. The myoclonic jerks are brief (1–3 s) and primarily involve the upper extremities and head, with less frequent involvement of the lower limbs. Additional manifestations include head nodding, upward eye rolling, and eyelid blinking. No other seizure type co-occurs in MEI, although the myoclonic events may be preceded by prior febrile seizures [79]. The jerks may be isolated or clustered, and tend to predominate during drowsiness or slow-wave sleep, though they may also arise spontaneously [80]. Recognized MEI variants include: (a) reflex myoclonic epilepsy of infancy (RMEI), triggered by unexpected tactile or auditory stimuli [81]; (b) photosensitive MEI, induced by photic stimulation; (c) nocturnal MEI, occurring exclusively during sleep [82]; and (d) familial infantile myoclonic epilepsy with autosomal recessive inheritance. No consensus exists on whether RMEI should be classified separately from classical MEI [80,83]. Caraballo et al. [84] recruited 102 infants with BMEI: clinical features encompassed myoclonus, spasms, brief tonic contractions, shuddering, atonia or negative myoclonus, and multiple motor phenomena. Episodes were predominantly observed during wakefulness, were recurrent several times daily, and occurred in clusters in nearly half of the patients. Interictal EEG is typically normal during both wakefulness and sleep. Paroxysmal slowing in central areas has been reported. Under photic stimulation, generalized spike-wave or polyspike-wave discharges may be recorded during drowsiness or sleep in approximately 20% of patients. In a subsequent series of 38 infants with MEI reported by Caraballo et al. [85], 12 had reflex myoclonus—10 triggered by tactile stimuli and 2 by noise and light; interictal EEG showed generalized spike-wave, polyspike, and polyspike-wave paroxysms, and was normal in 12 patients. At last follow-up, 32 patients had normal neurological and neuropsychological evaluations, two had significant cognitive impairment (IQ 60 and 63), and four had significant learning impairment, including two with ADHD.

Zuberi and O’Regan [86] reviewed the existing literature and six additional cases, concluding that long-term seizure outcomes are generally favorable with sodium valproate; however, cognitive difficulties may be present in approximately one-third of children with MEI at long-term follow-up, while no cognitive impairment was found in RMEI cases. Among seven patients with extended follow-up reported by Prats-Viñas et al. [87], three showed unfavorable intellectual and behavioral trajectories, and three developed generalized seizures during follow-up. Long-term neuropsychological outcomes in seven patients with BMEI were assessed by Mangano et al. [88]: 86% presented with neuropsychological and intellectual difficulties, including fine motor skill deficits, attention deficits, language impairment, and learning disorders.

A follow-up study by Domínguez-Carral et al. [89] in ten BMEI patients found that 60% achieved seizure freedom with valproate; IQ scores ranged from 74 to 93, with three patients in the borderline intelligence range and six in the medium-to-low range. ADHD was present in nearly all patients assessed. Gene mutations specifically associated with MEI are infrequent. Using next-generation sequencing for idiopathic epilepsy screening, Campostrini et al. [90] identified the heterozygous variant p.Arg550Cys (c.1648C>T) in *HCN4* in two brothers affected by BMEI. In a large epilepsy gene study, Wang et al. [91] found *TBCID24* involvement in a family with infantile myoclonic epilepsy. Valproic acid remains the treatment of choice for MEI, with clonazepam and levetiracetam also showing favorable results.

### 3.8. Benign Genetic Epilepsies: From Molecular Pathogenesis to Clinical Phenotypes

Benign genetic epilepsies represent a continuum of neurological disorders in which the genetic landscape shapes both fundamental neuronal function and the dynamics of cortical network development [92,93]. This interplay determines a wide spectrum of phenotypes, ranging from clinically self-limited forms to severe encephalopathic presentations associated with significant neurological disability. From a pathogenetic standpoint, most monogenic epilepsies involve ion channels, synaptic receptors, or transporters that directly influence neuronal excitability, the excitatory/inhibitory (E/I) balance, and the maturation of cortical circuits. Genes most commonly implicated include *SCN1A*, *SCN2A*, *KCNQ2*, *GRIN2A*, *SLC2A1*, and *SLC6A1*, alongside variants participating in metabolic pathways or synaptic transmission—such as *GABRA1*, *GABRG2*, and *STXBP1* [15,20,92].

In self-limited focal epilepsies, the causative genetic variants typically produce transient functional perturbations in neuronal excitability without permanently disrupting brain maturation [94,95]. For example, *KCNQ2* and *SCN2A* mutations have been found in self-limited neonatal-infantile forms; in these cases, the potassium channel Kv7.2 or the sodium channel Nav1.2 undergoes functional shifts that heighten neuronal excitability during a critical developmental window but do not interfere with long-term cortical organization, thereby allowing spontaneous seizure remission in many patients [92]. *GRIN2A*, encoding an NMDA receptor subunit, is associated with benign focal phenotypes, likely through subtle alterations in glutamatergic transmission that affect synaptic plasticity without inducing widespread cortical network dysfunction [96]. *SCN1A* variants may manifest either as benign focal epilepsy or within the broader GEFS+ spectrum, reflecting phenotypic complexity dependent on the specific nature and location of the mutation within the gene [97,98]. The identification of underlying channelopathies has progressively redirected the therapeutic paradigm toward precision medicine. In patients carrying *KCNQ2* or *SCN2A* gain-of-function mutations, sodium channel blockers such as oxcarbazepine, carbamazepine, and phenytoin have demonstrated superior efficacy over phenobarbital, achieving faster seizure control and, in some cases, improved neurodevelopmental outcomes. Early genetic testing is therefore recommended in neonates with refractory seizures, as antiseizure medication selection should ideally be guided by the underlying molecular mechanism rather than by empirical drug escalation.

Taken together, the evidence reviewed here highlights the substantial heterogeneity underlying the SELFEs spectrum and the limitations of considering these syndromes as uniformly “benign.” Although most patients experience favorable seizure outcomes, prognostic variability remains considerable, particularly regarding cognitive and neuropsychiatric trajectories. The distinction between self-limited epilepsies and developmental and epileptic encephalopathies may therefore represent a continuum rather than a strict categorical separation, especially in disorders associated with ion-channel dysfunction. Furthermore, unresolved classification issues persist, including the debated inclusion of GEFS+ and MEI within the SELFE group and the positioning of POLE within reflex epilepsies. Current literature also underscores the need for longitudinal studies integrating electroclinical, neuropsychological, and genetic data in order to better identify predictors of atypical evolution and treatment response. Future advances in molecular diagnostics and precision medicine may further refine syndrome classification and support individualized therapeutic strategies aimed not only at seizure control but also at preservation of neurodevelopmental outcomes.

### 3.9. Evolution and Treatment

Vikin et al. [99] analyzed a birth cohort of 114,000 children born between 1999 and 2009 with epilepsy syndromes classified according to epilepsy type, with the aim of characterizing the course of epileptic episodes. Overall, 78% of the cohort achieved seizure freedom. Outcomes were particularly favorable in children with focal epilepsy (84%), while the lowest rates of seizure freedom were observed in combined generalized and focal epilepsy (26%). The best outcomes were documented in self-limited focal epilepsies, childhood absence epilepsy, and self-limited infantile syndromes.

The appropriate management of children with self-limited focal epilepsy remains a subject of debate. Given that these disorders are generally characterized by a favorable clinical course, antiseizure treatment is not always considered necessary, and the potential adverse effects of medications on the developing brain must be carefully weighed. Conversely, withholding therapy carries the risk of ongoing seizure activity with negative psychosocial consequences for the child in both school and social settings. For these reasons, treatment decisions in SELFE should be individualized, considering both the syndrome type and the specific child’s circumstances. Key factors to consider include the age of the patient—since neonates and infants are particularly vulnerable to drug-related adverse effects—as well as EEG findings, seizure type, and the duration, severity, and frequency of episodes, along with the clinical and EEG response to therapy. Seizure suppression can have a meaningful positive psychological impact on both the child and the family, including at school and in peer relationships. For SELFEs, following a careful assessment of the indications for pharmacological treatment, the following antiseizure management (ASM) strategies are recommended: for SeLFNIE, phenobarbital as first-line [19], with phenytoin, levetiracetam, midazolam, or lidocaine as second-line options; for broader SELFEs, lamotrigine or levetiracetam as first-line agents, though carbamazepine, oxcarbazepine, valproate, and zonisamide are also in use.

## 4. Limitations

This review has several limitations that should be acknowledged. In addition, given the nature of the review, no formal quality or risk-of-bias assessment of the included studies was performed, limiting the ability to weigh the strength of the evidence. The included literature is highly heterogeneous, encompassing case reports, retrospective studies, and cohort analyses with variable diagnostic criteria and evolving classifications of SELFEs, which may affect the comparability of findings. Furthermore, the ongoing refinement of the International League Against Epilepsy framework and the lack of consensus regarding the inclusion of certain syndromes (e.g., GEFS+ and MEI) introduce additional variability in interpretation. The genetic landscape of SELFEs is also rapidly evolving, and current knowledge may not fully capture emerging variants or genotype–phenotype correlations. Finally, the absence of quantitative synthesis precludes definitive conclusions regarding treatment efficacy or prognostic outcomes, and the results should therefore be interpreted as a qualitative mapping of the existing evidence rather than as a basis for clinical standardization.

## 5. Conclusions

SELFEs constitute a clinically relevant group of disorders on account of their notable frequency within the spectrum of pediatric neurological conditions, their generally favorable prognosis in the majority of cases, and the considerable psychological burden they place on both affected children and their families. Several unresolved issues remain within this group: (a) a subset of disorders classically considered to have a favorable course have been shown in a small proportion of patients to carry additional negative clinical implications; (b) there is no universal agreement on which conditions should be classified as SELFEs, as some previously included syndromes have demonstrated complicated clinical courses; the most recent consensus recognizes the following disorders as SELFEs: self-limited familial and non-familial neonatal-infantile convulsions, self-limited epilepsy with autonomic seizures, self-limited epilepsy with centrotemporal spikes, childhood occipital visual epilepsy, and photosensitive occipital lobe epilepsy [15]; and (c) treatment modalities must be individualized according to the specific syndrome, with primary consideration given to the duration, intensity, and frequency of seizures, as well as the broader impact on the child and family.

This review has examined several key clinical dimensions pertaining to this group of disorders. Emerging clinical observations and expanding genetic data continue to enhance our understanding of SELFEs and hold promise for further improvements in diagnosis, counseling, and personalized management.

## Figures and Tables

**Table 1:** Summary of the main clinical features of Self-Limited Focal Epilepsies in Childhood.

Syndrome	Age of Onset	Key Clinical Features	Interictal EEG	Gene Mutations	Spontaneous Remission
SeLFNIE	2–5 day of life (neonatal); first weeks–months (infantile)	Focal motor tonic/clonic seizures in clusters, alternating sides; apnea, cyanosis	Normal background; focal temporal/posterior discharges	*KCNQ2*, *KCNQ3*, *SCN2A*, *SCN8A*, *PRRT2*	6 weeks–24 months of age
SeLECTS	3–13 years (peak 9–10 yrs)	Brief hemifacial motor seizures; hypersalivation, drooling; predominantly nocturnal	Unilateral centrotemporal high-amplitude spikes, increased in sleep	*KCNQ2*, *KCNQ3*, *ELP4*, *GRIN2A*	Typically by adolescence
SeLEAS	1–14 years (peak 3–6 yrs)	Nocturnal seizures; tonic eye deviation, vomiting, pallor, ictal syncope; possible autonomic status epilepticus	Multifocal high-voltage spikes/slow waves, predominantly occipital	None identified (polygenic)	~82.9% seizure-free within 2 years from onset
COVE	1–18 years (peak 8–9 yrs)	Brief visual hallucinations, ictal blindness, post-ictal headache; mainly in awake state	Occipital spike-wave paroxysms; fixation-off sensitivity	Polygenic (*GABRA1*, *GABRG2* in familial cases)	77% achieve seizure freedom; EEG normalizes in 85% by late adolescence
POLE	4–17 years (mean 11 yrs)	Photic-induced visual seizures (colored spots, hallucinations, visual blurring); strong female predominance	Occipital spikes and waves; photoparoxysmal response	Polygenic	Remission in most cases by puberty
GEFS+	Infancy to childhood (variable)	Febrile seizures ± afebrile seizures; broad spectrum: absence, myoclonic, atonic, GTCS	Normal background; generalized discharges	*SCN1A*, *SCN1B*, *KCNT1*, *CACNA1A*, *GABRG2*, *ABRA1*, *GABRB2*, *ABRB3*	Tends to remit by late childhood/puberty
MEI	First 3 years of life	Brief (1–3 s) myoclonic jerks of upper limbs and head; triggered by drowsiness or sensory stimuli	Normal interictal EEG; generalized spike-wave under photostimulation in ~20%	*HCN4*, *TBCID24* (rare)	Variable; seizure control usually achieved with treatment

## Data Availability

No new data were created or analyzed in this study.

## Supplementary Materials

The following supporting information can be downloaded at: https://www.mdpi.com/article/10.3390/pediatric18030074/s1, Supplementary File. The PRISMA 2020 Checklist [100].

## Abbreviations

The following abbreviations are used in this manuscript:

BFIS	Benign familial infantile seizures
ILAE	International League Against Epilepsy
SeLE	Self-limited epilepsy syndrome
SeLFNE	Self-limited (familial) neonatal epilepsy
SeLFNIE	Self-limited neonatal-infantile epilepsy
SeLIE	Self-limited (familial) infantile epilepsy
GEFS+	Genetic epilepsy with febrile seizures plus
MEI	Myoclonic epilepsy of infancy
SeLECTS	Self-limited epilepsy with centrotemporal spikes
SeLEAS	Self-limited epilepsy with autonomic seizures
COVE	Childhood occipital visual epilepsy
POLE	Photosensitive occipital lobe epilepsy
SeLFE	Self-limited focal epilepsies
BECT	Benign epilepsy with centrotemporal spikes
BRE	Benign rolandic epilepsy
COE-G	Childhood occipital epilepsy-Gastaut
RMEI	Reflex myoclonic epilepsy of infancy
PMEI	Photosensitive myoclonic epilepsy
FMEI	Familial infantile myoclonic epilepsy
ASM	Antiseizure management
